# Taxonomic novelties in *Magnolia*-associated pleosporalean fungi in the Kunming Botanical Gardens (Yunnan, China)

**DOI:** 10.1371/journal.pone.0235855

**Published:** 2020-07-13

**Authors:** Dhanushka N. Wanasinghe, Nalin N. Wijayawardene, Jianchu Xu, Ratchadawan Cheewangkoon, Peter E. Mortimer

**Affiliations:** 1 CAS Key Laboratory for Plant Biodiversity and Biogeography of East Asia (KLPB), Kunming Institute of Botany, Chinese Academy of Science, Kunming, Yunnan, China; 2 Department of Entomology and Plant Pathology, Faculty of Agriculture, Chiang Mai University, Chiang Mai, Thailand; 3 World Agroforestry, East and Central Asia, Kunming, Yunnan, China; 4 Center for Mountain Futures, Kunming Institute of Botany, Honghe County, Yunnan, China; 5 Center for Yunnan Plateau Biological Resources Protection and Utilization, College of Biological Resource and Food Engineering, Qujing Normal University, Qujing, Yunnan, China; 6 Innovative Agriculture Research Center, Faculty of Agriculture, Chiang Mai University, Chiang Mai, Thailand; 7 Center of Excellence in Microbial Diversity and Sustainable Utilization, Chiang Mai University, Chiang Mai, Thailand; Beijing Forestry University, CHINA

## Abstract

This paper represents the first article in a series on Yunnanese microfungi. We herein provide insights into *Magnolia* species associated with microfungi. All presented data are reported from the Kunming Botanical Gardens. Final conclusions were derived from the morphological examination of specimens coupled with phylogenetic sequence data to better integrate taxa into appropriate taxonomic ranks and infer their relationships. *Shearia formosa*, the type species of *Shearia*, lacks type material, and its phylogenetic position accordingly remains unresolved. A fresh collection of *Shearia formosa*, obtained from *Magnolia denudata* and *M*. *soulangeana* in China, therefore, designated a neotype for stabilizing the application of the species and/or genus name. Phylogenetic analyses of a combined DNA data matrix containing SSU, LSU, RPB2 and TEF loci of representative Pleosporales revealed that the genera *Crassiperidium*, *Longiostiolum* and *Shearia* are a well-defined monophylum. It is recognized as the family Longiostiolaceae and strongly supported by Bayesian and Maximum Likelihood methods. Its members are characterized by immersed to semi-immersed, globose to subglobose ascomata with a central, periphysate ostiole, a peridium composed of rectangular to polygonal cells, cylindrical to clavate asci, broadly fusiform, hyaline to pale brown ascospores, a coelomycetous asexual morph with pycnidial conidiomata, enteroblastic, annellidic, ampulliform, doliiform or cylindrical conidiogenous cells and cylindrical to fusiform, transverse and sometimes laterally distoseptate conidia without a sheath or with a basal lateral sheath. *Nigrograna magnoliae* sp. nov. is introduced from *Magnolia denudata* with both asexual and sexual morphs. We observed the asexual morph of *Brunneofusispora sinensis* from the culture and therefore amended the generic and species descriptions of *Brunneofusispora*.

## Introduction

Located in Southwestern China, Yunnan is renowned for harboring one of the botanically richest and most diverse terrestrial regions on Earth [1]. Yunnan represents >50% of China’s overall floristic diversity and accounts for 18,000 vascular plant species [2], with high levels of endemism [3]. A highly variable climate and flourishing vegetation facilitate rapid fungal growth, reproduction and speciation. Based on its high level of plant diversity, it is estimated that approximately 104,000 fungal species may exist in Yunnan [4].

Conversely, among these fungal encounters, ascomycetes are being neglected compared to the level of research conducted on basidiomycetes in Yunnan Province. Owing to their abundance in all ecosystems, ascomycetous taxa simply cannot be overlooked in any region. Most taxa of ascomycetes are plant-associated fungi that can be pathogens, endophytes, saprobes or epiphytes across a wide range of hosts in terrestrial as well as aquatic habitats. Microfungi contribute both positively and negatively to human and economic well-being. As pathogens, they pose a threat to agriculture [5], and the rapid identification of potentially problematic species and accurate prediction of their behavior will facilitate the adoption of proper mitigation and phytosanitary measures. Given their ubiquitous nature, additional taxonomic knowledge are prerequisites to understanding the biological and environmental significance of ascomycetes. Supporting this obligation, the CAS Key Laboratory for Plant Biodiversity and Biogeography of East Asia (KLPB) has begun to study plant-based ascomycetes in Yunnan Province. The current study represents the first in a series comprising a taxonomic circumscriptive project of microfungi in Yunnan Province. It accounts for a group of ascomycetes recovered from the twigs of *Magnolia denudata* and *M*. *soulangeana* in the East Garden of Kunming Botanical Garden (Kunming, Panlong District). This garden functions as an ex-situ conservation of plants from Southwest China, particularly focusing on the conservation of endangered, endemic and economically important plant species native to the Yunnan Plateau and the southern Hengduan Mountains [6]. Based on morphology and multi-gene phylogenetic evidences of the collected ascomycetes, we characterized a neotype, a new species and a new host record in the order Pleosporales.

## Materials and methods

### Isolates and specimens

Fresh fungal materials were collected from twigs of *Magnolia denudata* and *M*. *soulangeana* in the East Garden of Kunming Botanical Garden (Yunnan Province, China) during both dry (February) and wet (August) seasons. Kunming Botanical Garden is in the central part of Yunnan Province in the city of Kunming, located in a plateau basin. The wet and dry seasons are distinct in Kunming, and precipitation is concentrated from May to October, accounting for about 85% of the annual precipitation [7]. The dry seasons is from November to April, the rainfall of accounts for only about 15% of total annual rainfall [7]. The Garden is located at 25°07’004.9”–25°08’054.8”N, 102°44’015.2”–102°44’047.3”E at an elevation of 1914–1990 m above sea level, and has an annual average rainfall of 1006.5 mm, an annual average temperature of 14.7 °C and an annual average relative humidity of 73% [6]. The collected specimens were brought to the laboratory in Zip lock plastic bags. Samples were examined with an Olympus SZ61 Series microscope. Single spore isolation was carried out following the method described in [8]. Germinated spores were individually transferred to potato dextrose agar (PDA) plates and grown at 20 °C in the daylight. Isolates including accession numbers of gene sequences are listed in are listed in Suppl. material 1: Table 1. Isolates listed as MFLUCC and KUMCC are maintained in the Culture Collection of Mae Fah Luang University, Chiang Rai, Thailand and Kunming Institute of Botany Culture Collection, China, respectively. Specimens have been deposited in the Mae Fah Luang University (MFLU) Herbarium, Chiang Rai, Thailand. Faces of Fungi and Index Fungorum numbers are provided as outlined in [9] and [10].

**Table 1:** Genes/loci used in the study with PCR primers, references and protocols.

Locus ^a^	Primers	PCR: thermal cycles: ^b^ (Annealing temp. in bold)	References
ITS	ITS5	(95 °C: 30 s, **55** °C:50 s, 72 °C: 90 s) × 35 cycles	[11]
	ITS4		
LSU	LR0R	(95 °C: 30 s, **55** °C:50 s, 72 °C: 90 s) × 35 cycles	[12, 13]
	LR5		
SSU	NS1	(95 °C: 30 s, **55** °C:50 s, 72 °C: 90 s) × 35 cycles	[11]
	NS4		
RPB2	fRPB2-5f	(94 °C: 60 s, **58** °C: 60 s, 72 °C: 90 s) × 40 cycles	[14]
	fRPB2-7cR		
TEF	EF1-983F	(95 °C: 30 s, **55** °C:50 s, 72 °C: 90 s) × 35 cycles	[15, 16]
	EF1-2218R		

^a^ ITS: Part of rDNA 18S (3' end), the first internal transcribed spacer (ITS1), the 5.8S rRNA gene, the second ITS region (ITS2), and part of the 28S rRNA (5' end); LSU: Large subunit (28S); SSU: Small subunit rDNA (18S); RPB2: RNA polymerase II second largest subunit; TEF: translation elongation factor 1-alpha gene

^b^ All the PCR thermal cycles include Initiation step of 95 °C: 5 min, and final elongation step of 72 °C: 10 min and final hold at 4 °C

### Morphological observations

In hand sections of the ascomata/ conidiomata, which were mounted in distilled water, the following characteristics were evaluated: ascomata/ conidiomata diameter, height, colour and shape; width of peridium; height and diameter of ostioles. Length and width (at the widest point) of asci, ascospores, conidiophores and conidia were measured. Images were captured with a Canon EOS 600D digital camera fitted to a Nikon ECLIPSE Ni compound microscope. Measurements were made with the Tarosoft (R) Image Frame Work program and images used for figures processed with Adobe Photoshop CS5 Extended version 10.0 software (Adobe Systems, USA).

### DNA extraction, PCR amplifications and sequencing

Mycelia for DNA extraction from each isolate were grown on PDA for 3–4 weeks at 20 °C and total genomic DNA was extracted from approximately 150 ± 50 mg axenic mycelium scraped from the edges of the growing culture. Mycelium was ground to a fine powder with liquid nitrogen and DNA extracted using the Biospin Fungus Genomic DNA Extraction Kit-BSC14S1 (BioFlux, P.R. China) following the instructions of the manufacturer. DNA to be used as template for PCR were stored at 4 °C for use in regular work and duplicated at -20 °C for long term storage.

DNA sequence data was obtained from the partial sequences of three ribosomal and two protein coding genes. The genes, primers, references and PCR protocols are summarized in Table 1. Polymerase chain reaction (PCR) was carried out in a volume of 25 μl which contained 12.5 μl of 2 × Power Taq PCR MasterMix (Bioteke Co., China), 1 μl of each primer (10 μM), 1 μl genomic DNA and 9.5 μl deionized water. The amplified PCR fragments were sent to a commercial sequencing provider (BGI, Ltd Shenzhen, P.R. China). The nucleotide sequence data acquired were deposited in GenBank.

### Molecular phylogenetic analyses

#### Sequencing and sequence alignment

Sequences generated from different primers of the five genes were analysed with other sequences retrieved from GenBank (see S1 Table). Sequences with high similarity indices were determined from a BLAST search to find the closest matches with taxa in Pleosporales, and from recently published data [17, 18, 19]. The multiple alignments of all consensus sequences, as well as the reference sequences were automatically generated with MAFFT v. 7 (http://mafft.cbrc.jp/alignment/server/index.html; [20, 21]), and were improved manually when necessary using BioEdit v. 7.0.5.2 [22].

#### Phylogenetic analyses

The single-locus data sets were examined for topological incongruence among loci for members of the Pleosporales. The conflict-free alignments were concatenated into a multi-locus alignment that was subjected to maximum-likelihood (ML) and Bayesian (BI) phylogenetic analyses. The evolutionary models for Bayesian analysis and maximum-likelihood were selected independently for each locus using MrModeltest v. 2.3 [23] under the Akaike Information Criterion (AIC) implemented in both PAUP v. 4.0b10. GTR+I+G model is resulted in each locus for Bayesian analysis and maximum-likelihood by AIC in MrModeltest as the best-fit model.

**Fig 1:**
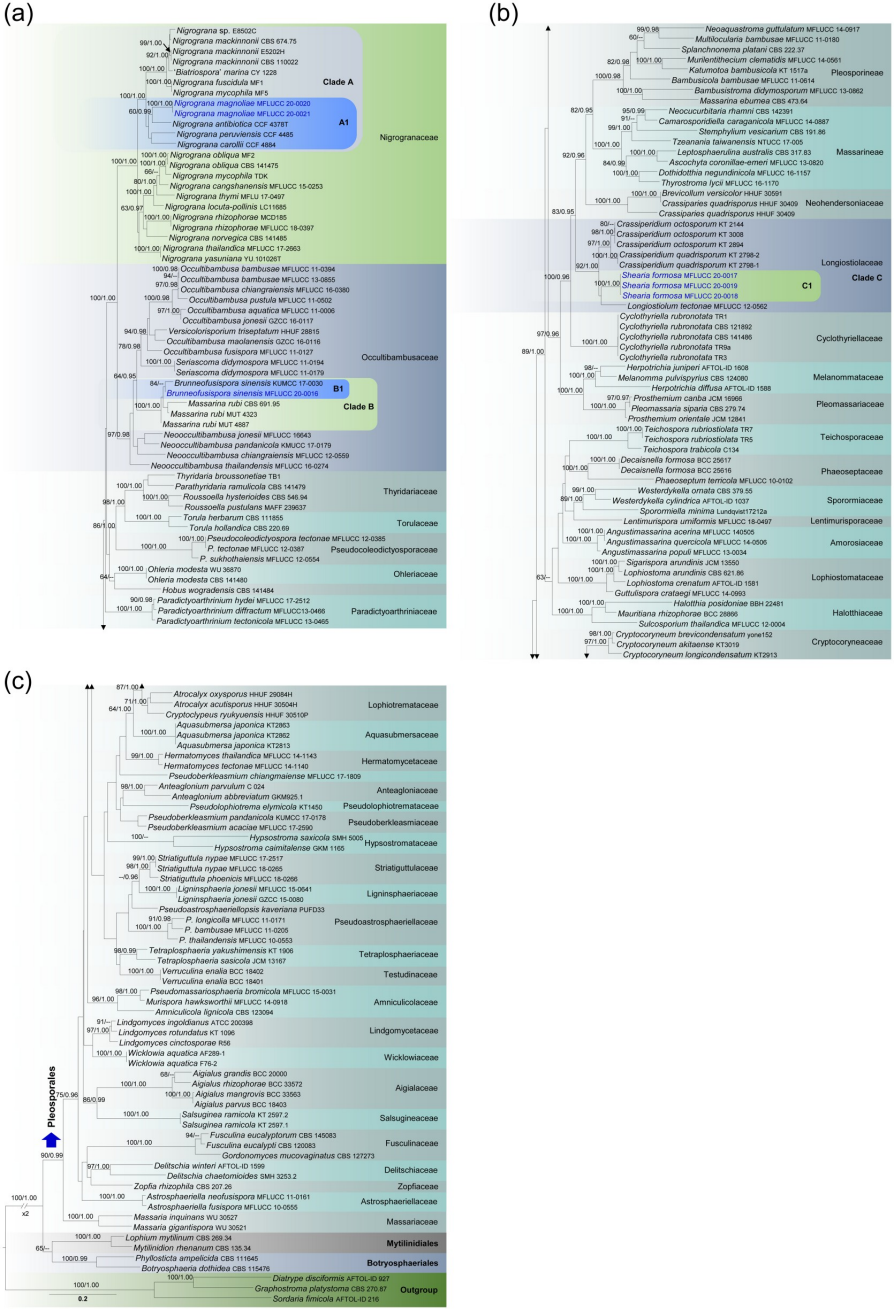
RAxML tree based on a combined dataset of partial SSU, LSU, RPB2 and TEF sequence analyses. Bootstrap support values for ML equal to or greater than 60% and Bayesian posterior probabilities (BYPP) equal to or greater than 0.95 are shown as ML/BI above the nodes. The new isolates are blue. The scale bar represents the expected number of nucleotide substitutions per site.

Bayesian analysis was conducted with MrBayes v. 3.1.2 [24] to evaluate Bayesian posterior probabilities (BYPP) [25, 26] by Markov Chain Monte Carlo sampling (BMCMC). GTR+I+G was used in the command. Six simultaneous Markov chains were run for 2,000,000 generations and trees were sampled every 200th generation. The distribution of log-likelihood scores was examined to determine stationary phase for each search and to decide if extra runs were required to achieve convergence, using the program Tracer 1.5 [27]. First 20% of generated trees were discarded and remaining 80% of trees were used to calculate posterior probabilities of the majority rule consensus tree. BYPP greater than 0.95 are given above each node (Fig 1).

Maximum likelihood trees were generated using the RAxML-HPC2 on XSEDE (8.2.8) [28, 29] in the CIPRES Science Gateway platform [30] using GTR+I+G model of evolution. Maximum likelihood bootstrap values (ML) equal or greater than 70% are given above each node (Fig 1).

Phylograms were visualized with FigTree v1.4.0 program [31] and reorganized in Microsoft power point (2007) and Adobe Illustrator^®^ CS5 (Version 15.0.0, Adobe^®^, San Jose, CA). The finalized alignment and tree were deposited in TreeBASE, submission ID: 25920 (http://purl.org/phylo/treebase/phylows/study/TB2:S25920).

## Compliance with ethical standards

The authors declare that there is no conflict of interest (financial or non-financial) and agree to the submission of this paper. The authors also confirm that the fieldwork did not involve endangered or protected species and no specific permissions were required for these locations as the land belongs to the Kunming Institute of Botany, which is the first affiliated institution on this paper.

## Nomenclature

The electronic version of this article in Portable Document Format (PDF) in a work with an ISSN or ISBN will represent a published work according to the International Code of Nomenclature for algae, fungi, and plants, and hence the new names contained in the electronic publication of a PLOS ONE article are effectively published under that Code from the electronic edition alone, so there is no longer any need to provide printed copies.

In addition, the new name contained in this work has been submitted to Index Fungorum from where they will be made available to the Global Names Index. The unique Index Fungorum number can be resolved and the associated information viewed through any standard web browser by appending the Index Fungorum number contained in this publication to the prefix www.indexfungorum.org/. The online version of this work is archived and available from the following digital repositories: PubMed Central, LOCKSS.

## Results

### Phylogenetic analysis

The concatenated dataset (SSU, LSU, RPB2 and TEF loci) consisted of 180 strains including the new taxa proposed in this study, with *Diatrype disciformis* (AFTOL-ID 927), *Graphostroma platystoma* (CBS 270.87) and *Sordaria fimicola* (AFTOL-ID 216) as the out-group taxa. Topologies of trees (ML and BI) derived from analyses of single gene dataset were compared and the overall tree topology was congruent to those obtained from the combined dataset.

The RAxML analysis of the combined dataset yielded a best scoring tree (Fig 1) with a final ML optimization likelihood value of -80641.514118. The matrix had 2357 distinct alignment patterns, with 24.7% of undetermined characters or gaps. Parameters for the GTR + I + G model of the combined LSU, SSU, RPB2 and TEF were as follows: Estimated base frequencies; A = 0.248843, C = 0.245092, G = 0.273138, T = 0.232926; substitution rates AC = 1.416663, AG = 4.473370, AT = 1.440148, CG = 1.224282, CT = 9.886557, GT = 1.000; proportion of invariable sites I = 0.46547; gamma distribution shape parameter α = 0.580812. Comparison of the alignment properties and nucleotide substitution models are provided in Table 2. The Bayesian analysis resulted in 10001 trees after 2000000 generations. The first 1000 trees, representing the burn-in phase of the analyses, were discarded, while the remaining 9001 trees were used for calculating posterior probabilities in the majority rule consensus tree.

**Table 2:** Comparison of alignment properties of genes and nucleotide substitution models used in Pleosporales phylogenetic analysis.

	SSU	LSU	RPB2	TEF
Alignment strategy (MAFFT v. 7)	G-INS-1	G-INS-1	G-INS-1 +manual	G-INS-1 +manual
Nucleotide substitution models for Bayesian analysis (determined by MrModeltest	GTR+I+G	GTR+I+G	GTR+I+G	GTR+I+G

Forty-one families and two suborders (Massarineae, Pleosporineae) in Pleosporales are represented in the phylogenetic tree (Fig 1). The topologies resulting from the ML and BI analyses (Fig 1) are generally congruent with the results reported by [19] and other large-scale phylogenies of Dothideomycetes e.g. [32]. Multigenic (Fig 1) analyses agree to support significantly the monophyletic origin of every interleaved family and the two suborders in Pleosporales. Six newly isolated strains from this study (MFLUCC 20–0016, MFLUCC 20–0017, MFLUCC 20–0018, MFLUCC 20–0019, MFLUCC 20–0020 and MFLUCC 20–0021) constituted three lineages in Clade A, B and C (Fig 1). MFLUCC 20–0020 and MFLUCC 20–0021 grouped within Nigrogranaceae (Clade A, Fig 1) as a well-supported monophyletic clade with 100% ML 1.00 BYPP support (Fig 1). This is closely related to *Nigrograna antibiotica* (CCF 4378T), *N*. *carollii* (CCF 4884) and *N*. *peruviensis* (CCF 4485), but the corresponding affiliation is not statistically supported (Subclade A1, Fig 1). MFLUCC 20–0016 grouped in Occultibambusaceae (Clade B, Fig 1). MFLUCC 20–0016, *Brunneofusispora sinensis* (KUMCC 17 0030) and three strains labeled as ‘*Massarina rubi*’ (CBS 691.95 MUT 4323, MUT 4887) formed a strongly supported monophyletic association (Subclade B1, Fig 1) in Occultibambusaceae. Three of our newly isolated strains (MFLUCC 20–0017, MFLUCC 20–0018, MFLUCC 20–0019), *Crassiperidium quadrisporum* (KT 2798 1, KT 2798 2), *C*. *octosporum* (KT 3008, KT 2144, KT 2894) and *Longiostiolum tectonae* (MFLUCC 12 0562) formed an isolated, well-supported clade (92% ML and 1.00 PP, Clade C, Fig 1) within Pleosporales, which is regarded as Longiostiolaceae.

### Taxonomy

**Longiostiolaceae** Phukhams., Doilom & K.D. Hyde, Fungal Diversity 101: 43 (2020) *amended*

[urn:lsid:indexfungorum.org:names: IF557086]

Facesoffungi number: FoF 07215.

*Saprobic* in terrestrial habitats. **Sexual morph**: *Ascomata* solitary to gregarious, immersed to semi-immersed, globose to subglobose, with a central ostiole. *Ostiole* long, circular, central, sometimes periphysate. *Peridium* composed of rectangular to polygonal, pale brown to dark brown cells. *Hamathecium* comprising numerous, cellular, septate pseudoparaphyses. *Asci* 4–8-spored, bitunicate, fissitunicate, cylindrical to clavate, pedicellate. *Ascospores* mostly overlapping biseriate to 3-seriate, broadly fusiform, thick-walled, 1–10-septate, hyaline to pale brown, smooth-walled. **Asexual morph**: *Conidiomata* pycnidial, sometimes pseudostromatic, solitary to gregarious, immersed, globose to conical, unilocular, dark brown, ostiolate. *Ostiole* papillate ostiole, central, circular. *Conidiomata wall* composed of angular, pale brown to brown cells. *Conidiophores* absent or reduced to conidiogenous cells. *Conidiogenous cells* enteroblastic, annellidic, ampulliform, doliiform or cylindrical. *Conidia* cylindrical to fusiform, base truncate, with several transverse and sometimes laterally distoseptate, continuous, without a sheath or with a basal lateral sheath.

Type genus: *Longiostiolum* Doilom, Ariyaw. & K.D. Hyde

*Notes*: Clade C (Fig 1) comprises *Crassiperidium*, *Longiostiolum* and *Shearia*, which are phylogenetically highly supported and belong to Longiostiolaceae in the Pleosporales (Fig 1). [33] recently introduced Longiostiolaceae to accommodate *Crassiperidium* and *Longiostiolum*. In this study, *Shearia* has also proven to be a genus in Longiostiolaceae (Clade C1, Fig 1), and we hereby amend the family description in order to accommodate its morphological characteristics.

Longiostiolaceae has a close phylogenetic affinity to Cyclothyriellaceae in large-scale multi-gene phylogenetic analyses of Pleosporales. But they are not grouped in a monophyletic clade, rather forming discrete clades adjacent to each other. The asexual characteristics of this new family distinguish it from Cyclothyriellaceae. Cyclothyriellaceae has 1-celled conidia, whereas Longiostiolaceae has multi-celled conidia. Morphologically, *Crassiperidium* is most similar to *Pseudoasteromassaria* [34] in its cylindrical, multi-septate, hyaline conidia. However, their ascospores are different, and phylogenetically, both of them are not closely associated. *Shearia* shares some morphological resemblances to camarosporium-like and stegonsporiopsis-like taxa by its pycnidial conidiomata, holoblastic annellidic conidiogenous cells and distoseptate, pale pigmented conidia [35]. However, neither camarosporium-like nor stegonsporiopsis-like taxa are phylogenetically closely related to *Shearia*.

### Accepted genera in Longiostiolaceae

***Crassiperidium*** M. Matsum. & Kaz. Tanaka, Mycosphere 9(6): 1259 (2018)

Type species: *Crassiperidium octosporum* M. Matsum. & Kaz. Tanaka (2018)

*Notes*: [18] established the genus *Crassiperidium* to accommodate two new species, *C*. *octosporum* and *C*. *quadrisporum* from *Fagus crenata* in Japan. *Crassiperidium* is characterized by ‘globose to depressed globose ascomata with a well-developed ascomatal wall at the sides, clavate asci, broadly fusiform, hyaline ascospores, pycnidial conidiomata, and cylindrical, multi-septate, hyaline conidia produced by annellidic conidiogenous cells’ [18]. Phylogenetic analyses of multi-genes show that *Crassiperidium* is closely affiliated with Cyclothyriellaceae (Pleosporales, Dothideomycetes), but the exact familial placement was uncertain. Morphologically, *Crassiperidium* is similar to *Pseudoasteromassaria* by its ascomatal, pycnidial and conidial characteristics [18, 34]. Also, both are recorded from the same host plant (*Fagus crenata*). However, phylogenetically, *Pseudoasteromassaria* belongs to Latoruaceae and is not closely related to *Crassiperidium* (Fig 1). In addition, their ascospores and conidiogenous cells differ [18, 36].

***Longiostiolum*** Doilom, Ariyaw. & K.D. Hyde, Fungal Diversity 78: 55 (2016)

Type species: *Longiostiolum tectonae* Doilom, D.J. Bhat & K.D. Hyde, Fungal Diversity 78: 55 (2016)

*Notes*: *Longiostiolum* is introduced by [37] as a monotypic genus in the suborder Massarineae with *L*. *tectonae* as the type species. The genus is characterized by black, immersed to semi-immersed, uniloculate, globose to subglobose ascostromata with a long central ostiole and phragmosporous ascospores. *Longiostiolum* was an *incertae sedis* genus in Pleosporales and [38] listed this in Massarinaceae. In this study, *Longiostiolum tectonae* grouped sister to *Shearia* in Longiostiolaceae (Clade C, Fig 1).

***Shearia*** Petr., Annales Mycologici 22 (1–2): 180 (1924)

Type species: *Shearia formosa* (Ellis & Everh.) Petr., Sydowia 15 (1–6): 216 (1962)

*Notes*: [39] introduced the genus *Shearia* with *S*. *magnoliae* (Shear) Petr. (basionym *Camarosporium magnoliae* Shear 1902) as the type species. However, [40] regarded that *Stegonsporium formosa* Ellis & Everh. (1883) is the oldest name for this taxon and thus introduced the new name, *Shearia formosa* (Ellis & Everh.) Petr. (1962). [41], however, erroneously listed *Shearia magnoliae* as the type species of *Shearia*, although [42] accepted *Shearia formosa* as the type species. [35] illustrated *Shearia fusa* (Berk. & M.A. Curtis) M.E. Barr based on HHUF 30474 (on twigs of *Magnolia praecocissima* var. *borealis*), Japan.

[42] mentioned that *Pleomassaria magnoliae* Shear (1902) is the sexual morph of *Shearia formosa* (as *Camarosporium magnoliae*). [43] reported *Shearia fusa* from putative culture of *Pleomassaria maxima* Ellis & Everh. (current name: *Splanchnonema maximum* (Ellis & Everh.) M.E. Barr *fide* [44]. However, the asexual morph of *Pleomassaria maxima* was reported as *Shearia formosa* [45]. There are no sequence data available for *Pleomassaria maxima* (= *Splanchnonema maximum*) in GenBank, thus it is not possible to check its phylogenetic relationship with other taxa in Pleosporales. With the availability of molecular data, we have included *Pleomassaria siparia* (CBS 279.74) and *Splanchnonema platani* (CBS 222.37) in our phylogenetic analyses to represent *Pleomassaria* and *Splanchnonema*. However, they are not closely related to each other (Fig 1), whereas CBS 279.74 grouped in Pleomassariaceae and CBS 222.37 grouped in Massarineae.

During this study of microfungi from *Magnolia*, we recollect *Shearia fermosa* from Kunming. Below we illustrate and re-describe our new collection as a neo-type. In multi-gene analyses (Fig 1), *Shearia formosa* (MFLUCC 20–0017, MFLUCC 20–0018, MFLUCC 20–0019) groups in Longiostiolaceae.

***Shearia formosa*** (Ellis & Everh.) Petr., Sydowia 15 (1–6): 216 (1962) Fig 2.

**Fig 2:**
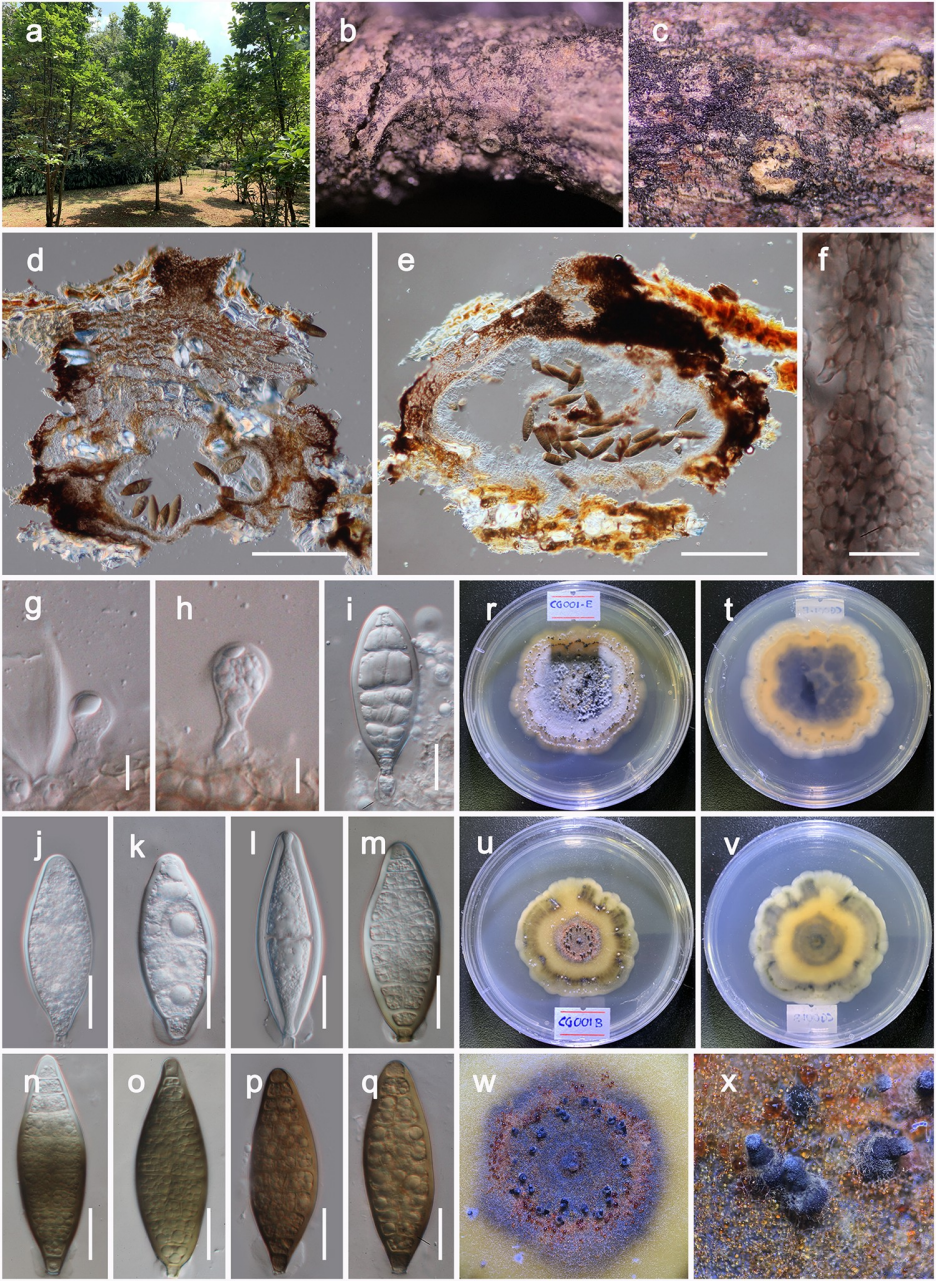
*Shearia formosa* (Neotype, MFLU 19–2368). a Habitat. b, c Conidiomata observed on host substrate. d, e Vertical section of conidiomata. f Cells of pycnidia wall. g–i Conidiogenous cells and developing conidia. j–q Conidia. r–v Colonies on PDA (t and v from the bottom). w, x Conidiomata on PDA. Scale bars: 200 μm (d, e), 20 μm (f, i, j–q), 10 μm (g, h).

≡ *Stegonsporium formosa* Ellis & Everh., Bull. Torrey bot. Club 10(7): 76 (1883)

= *Shearia magnoliae* (Shear) Petr., Annls mycol. 22(1/2): 180 (1924)

= *Camarosporium magnoliae* Shear, Bull. Torrey bot. Club 29: 455 (1902)

[urn:lsid:indexfungorum.org:names:339263]

Facesoffungi number: FoF 07747

*Saprobic* on dead and living twigs of *Magnolia* spp. **Sexual morph**: Undetermined. **Asexual morph**: *Conidiomata* 500–700 μm high, 600–900 μm diam. ($\bar{\text{x}}=636.4\times 795.1$ μm, n = 10), pseudostromatic, solitary to gregarious, immersed, peridermal to subperidermal, globose to conical, unilocular, dark brown, papillate ostiole, central, circular. *Conidiomata wall* 15–25 μm wide at the base, 40–70 μm wide at the sides outer layer composed of thick-walled, very dark brown occluded cells, lateral and basal walls composed of peridermal cells and thick-walled, brown cells of *textura angularis*. *Conidiophores* reduced to conidiogenous cells. *Conidiogenous cells* 8–12 μm long, 5–8 μm wide ($\bar{\text{x}}=9.9\times 6.4$ μm, n = 20), holoblastic, annellidic, ampulliform, doliiform or cylindrical, discrete, indeterminate, hyaline, often thick and smooth-walled. *Conidia* 70–95 μm × 24–30 μm ($\bar{\text{x}}=82.8\times 26.9$ μm, n = 30), fusiform, base truncate, apex obtuse, with several transverse and laterally distoseptate, continuous, smooth and thick-walled; initially enveloped in a gelatinous sheath, depressed at the apex, at maturity remaining as a basal lateral sheath.

Culture characteristics: When cultured on PDA, colonies reached up to 40 mm diam after 10 d at 18 °C with smooth and undulate edge, whitish at the beginning, becoming pale brown in center, yellowish green in median area, creamy margin, slightly raised, circular appearance, with sparse to moderate aerial mycelium on the surface with black, scattered conidiomata. On reverse, dirty green or brownish orange, with distinct zonation.

Known Distribution: *Magnolia* sp.: China [46; this study], *Magnolia kobus*: Japan [43, 47], *Magnolia grandiflora*, *Magnolia* sp.: USA [42, 48]

Material examined: CHINA, Yunnan Province, Heilongtan, Kunming Institute of Botany, 25.137711° N, 102.745185° E, on living branches of *Magnolia denudata* (Magnoliaceae), 02 February, 2019, D.N. Wanasinghe, DWCG001 (**neotype designated here**, MFLU 19–2368), ex-neotype living cultures MFLUCC 20–0019, KUMCC 20–0181, *ibid*. DWCG001E (MFLU 19–2369), living cultures MFLUCC 20–0018, KUMCC 20–0180, ibid. 19 August 2019, on living branches of *Magnolia soulangeana* DWCG001B (MFLU 20–0091), living cultures MFLUCC 20–0017, KUMCC 20–0182.

Notes: We could not find any herbarium materials for *Shearia formosa*, *Stegonsporium formosa* or *Camarosporium magnoliae*. There are three herbarium specimens were located in the herbarium of Royal Botanic Gardens (Kew, HerbIMI) for *Shearia viz*. IMI 200150 (1947), IMI 200273 (1904) and IMI 365970 (1904) as *S*. *magnoliae* from *Magnolia* spp. However, all three are labelled as *Shearia magnoliae* and none of them is regarded as type. Since the holotype of *Shearia formosa* seems to be lost or not elected, a neotype specimen (MFLU 19–2368) and an ex-neotype culture (MFLUCC 20–0019) are designated here to fix the use of the name. The descriptions and illustrations in [42] for *Shearia formosa* (from IMI 200150) are similar to MFLU 19–2368 which we describe herein (Fig 2).

**Nigrogranaceae** Jaklitsch & Voglmayr, Hyde, Stud. Mycol. 85: 54 (2016)

***Nigrograna*** Gruyter, Verkley & Crous, Stud. Mycol. 75: 31 (2012)

Type species: *Nigrograna mackinnonii* (Borelli) Gruyter, Verkley & Crous, Stud. Mycol. 75: 31 (2012)

Notes: The genus *Nigrograna* was introduced by [49] with *Nigrograna mackinnonii* (basionym: *Pyrenochaeta mackinnonii* Borelli) as the type species. Originally, *Pyrenochaeta mackinnonii* was reported from a mycetoma of a human. However, the later studies by [17, 50, 51, 52] supplemented more species associated with plants. [53] introduced *Nigrograna antibiotica*, *N*. *carollii*, *N*. *peruviensis* and *N*. *yasuniana* by synonymizing the endophytic *Biatriospora* species in [54]. There are 14 species listed in [10]. In this study, we introduce *Nigrograna magnoliae* as a new species which was collected from *Magnolia denudata* in Kunming, China.

**Fig 3:**
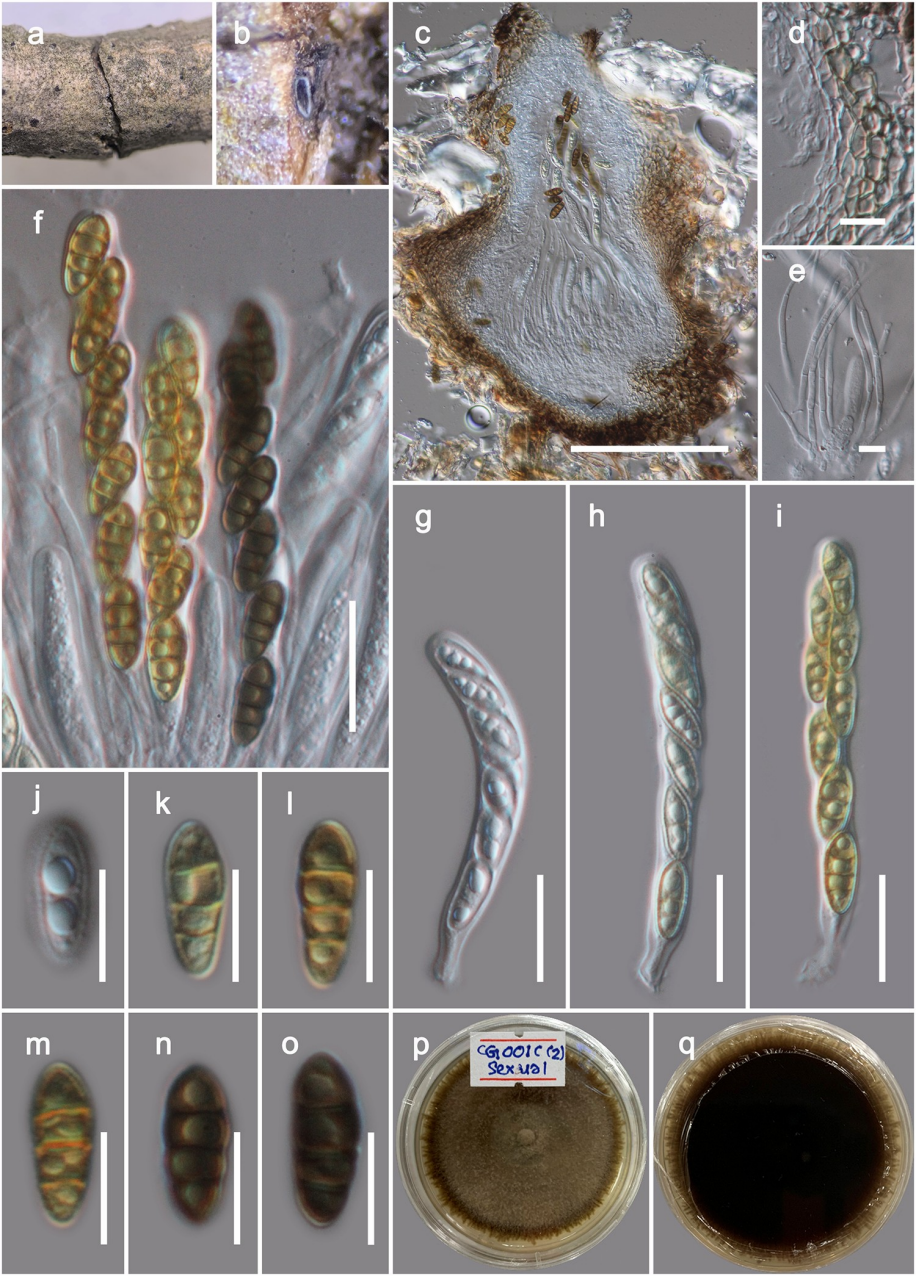
Sexual morph of *Nigrograna magnoliae* (MFLU 20–0092, Holotype). a, b Ascomata observed on host substrate. c Vertical sections through an ascoma. d Cells of peridium. e Pseudoparaphyses. f–i Asci. j–o Ascospores. p, q Colonies on PDA (q from the bottom). Scale bars: 100 μm (c), 20 μm (f–i), 10 μm (e, j–o).

**Fig 4:**
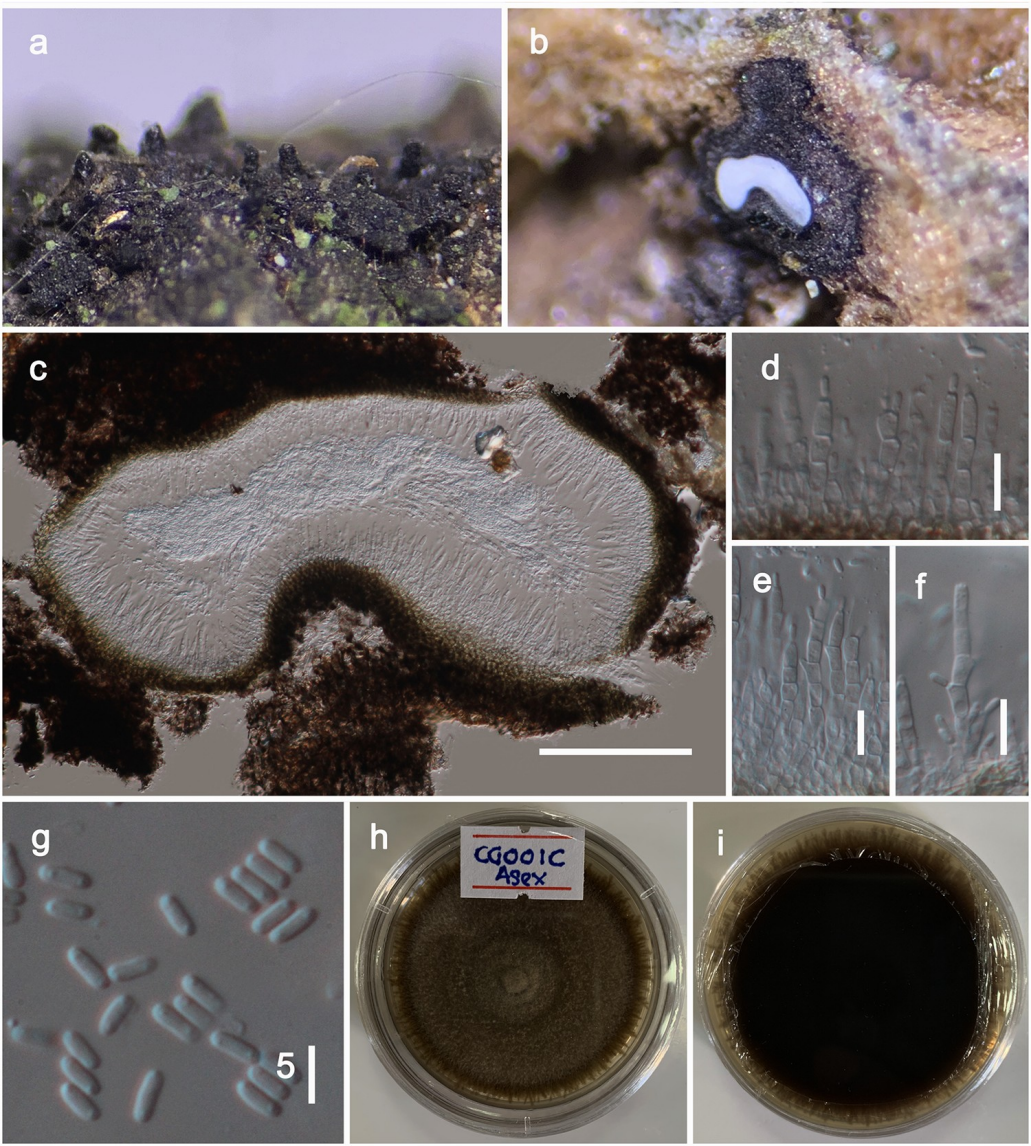
Asexual morph of *Nigrograna magnoliae* (MFLU 20–0092, Holotype). a, b Conidiomata observed on host substrate. c Vertical sections through a conidioma. d–f Conidiophores and phialides. g Conidia. h, i Colonies on PDA (i from the bottom). Scale bars: 100 μm (c), 10 μm (d–f), 5 μm (g).

***Nigrograna magnoliae*** Wanas. *sp*. *nov*. Figs 3 and 4

[urn:lsid:indexfungorum.org:names: 557331]

Facesoffungi number: FoF 06278

*Etymology*: The name of the species refers to the host genus, *Magnolia*.

*Saprobic* on dead aerial twigs of *Magnolia denudata*. **Sexual morph**: *Ascomata* 200–300 μm high, 150–220 μm diam. ($\bar{\text{x}}=243.7\times 186.2$ μm, n = 10), perithecioid, solitary or gregarious, immersed to erumpent through host tissue, subglobose or obpyriform, brown to dark brown, with an ostiole. *Ostiole* mostly central, brittle. *Peridium* composed of angular cells, outer layer, dark brown, thick-walled cells, inner layer, hyaline with thin-walled cells. *Hamathecium* composed of numerous, 2–3 μm wide, filamentous, septate pseudoparaphyses. *Asci* 60–100 × 8–12 μm (x = 74.2 × 9.5 μm, n = 20), 8-spored, bitunicate, fissitunicate, clavate to cylindric-clavate, short pedicellate, apically rounded, with a minute ocular chamber. *Ascospores* 12–16 × 5–6.5 μm (x = 14.1 × 5.7 μm, n = 30), overlapping uni to bi-seriate, ellipsoid, yellowish-brown to brown, 3-septate, guttulate. **Asexual morph** (on the natural host): Coelomycetous. *Conidiomata* 300–500 μm high, 200–300 μm diam. ($\bar{\text{x}}=414.9\times 217.1$ μm, n = 10), pseudostromatic, solitary to gregarious, immersed, peridermal to subperidermal, globose to conical, unilocular, dark brown, papillate ostiole, central, circular. *Conidiomata wall* 15–30 μm wide, outer layer composed of thick-walled, very dark brown occluded cells, inner layers composed with hyaline, thin cells of *textura angularis*. *Conidiophores*, branched at the base, septate, hyaline, straight or sinuous to slightly curved, with pegs along one or two sides and solitary phialides terminally. *Phialides* 10–30 μm long, 2–4 μm wide ($\bar{\text{x}}=18.6\times 3.4$ μm, n = 30), variable in shape, ampulliform-lageniform-subcylindrical, hyaline, smooth-walled. *Conidia* 3.5–5 μm × 1.3–1.7 μm ($\bar{\text{x}}=4.1\times 1.5$ μm, n = 30), oblong to cylindrical or allantoid, subhyaline to hyaline, 1-celled, containing 2 guttules, smooth.

*Culture characteristics*: On PDA, colonies reached up to 40 mm diam after 12 d at 18 °C. Colony dense, circular, slightly raised, surface smooth, with serrate edge, floccose, greenish grey at the center and brown towards margin from the top and reverse dark brown.

*Material examined*: CHINA, Yunnan Province, Heilongtan, Kunming Institute of Botany, 25.137711° N, 102.745185° E, on living branches of *Magnolia denudata* (Magnoliaceae), 02 February, 2019, D.N. Wanasinghe, DWCG001C (MFLU 20–0092), ex-type living cultures MFLUCC 20–0020, KUMCC 20–0178 (asexual), MFLUCC 20–0021, KUMCC 20–0179 (sexual).

*Notes*: The new fungus was collected from *Magnolia denudata* in Kunming. It can be labeled a typical *Nigrograna* taxa based on its ascomata, asci and ascospore characteristics. Phylogenetically it has a close affinity to *Nigrograna antibiotica*, *N*. *carollii* and *N*. *peruviensis*, nonetheless this relationship is statistically not supported (Subclade A1, Fig 1). These three species are reported as endophytes from Peru in the phloem of living *Ulmus laevis*, on living sapwood of wild *Hevea brasiliensis*, and on living sapwood of wild *Virola* sp. respectively [54]. All of these species are known only from their culture characteristics, precluding comparing the morphology of either sexual or asexual morph with our new collection. We were able to isolate both ascospores and conidia from the fruiting structures on the natural host. Both single spore isolation of ascospore and conidia formed identical culture morphologies on PDA (Figs 3p, 3q, 4h and 4i) and the DNA based sequences derived from those cultures were also similar in comparisons. Therefore, we introduce *Nigrograna magnoliae* sp. nov. providing both asexual and sexual morphs.

**Occultibambusaceae** D.Q. Dai & K.D. Hyde, Fungal Diversity 82: 25 (2016)

*Notes*: [55] introduced family Occultibambusaceae and currently it comprises five genera *viz*. *Brunneofusispora* S.K. Huang & K.D. Hyde, *Neooccultibambusa* Doilom & K.D. Hyde, *Occultibambusa* D.Q. Dai & K.D. Hyde, *Seriascoma* Phook., D.Q. Dai & K.D. Hyde and *Versicolorisporium* Sat. Hatak., Kaz. Tanaka & Y. Harada [56]. *Brunneofusispora* was introduced by [57] to accommodate *B*. *sinensis* and it was known only from its sexual morph. We collected *Brunneofusispora sinensis* from *Magnolia* and in this study, we report the coelomycetous asexual morph from the culture. Hence, we amend *Brunneofusispora* in order to accommodate its asexual morph.

***Brunneofusispora*** S.K. Huang & K.D. Hyde, Fungal Diversity 95: 36 (2019) *amended*

[urn:lsid:indexfungorum.org:names:555599]

Facesoffungi number: FoF04862

*Saprobic* on dead wood or twigs. **Sexual morph**. See [57]. **Asexual morph**. Coelomycetous. *Conidiomata* solitary, superficial, globose to sub-globose, unilocular, dark brown. *Conidiomata wall* composed of thick-walled, dark brown cells of *textura angularis*. *Conidiophores* reduced to conidiogenous cells. *Conidiogenous cells* enteroblastic, phialidic, ampulliform, doliiform or cylindrical, discrete, indeterminate, hyaline, smooth-walled. *Conidia* oblong to cylindrical, 1-celled, hyaline, smooth, guttulate.

Type species. *Brunneofusispora sinensis* S.K. Huang & K.D. Hyde

Notes: Based on the combined SSU, LSU, RPB2 and TEF sequence analyses of Pleosporales, one of our new strain (MFLUCC 20–0016) clusters with *Brunneofusispora sinensis* with strong bootstrap support (Subclade B1, Fig 1).

**Fig 5:**
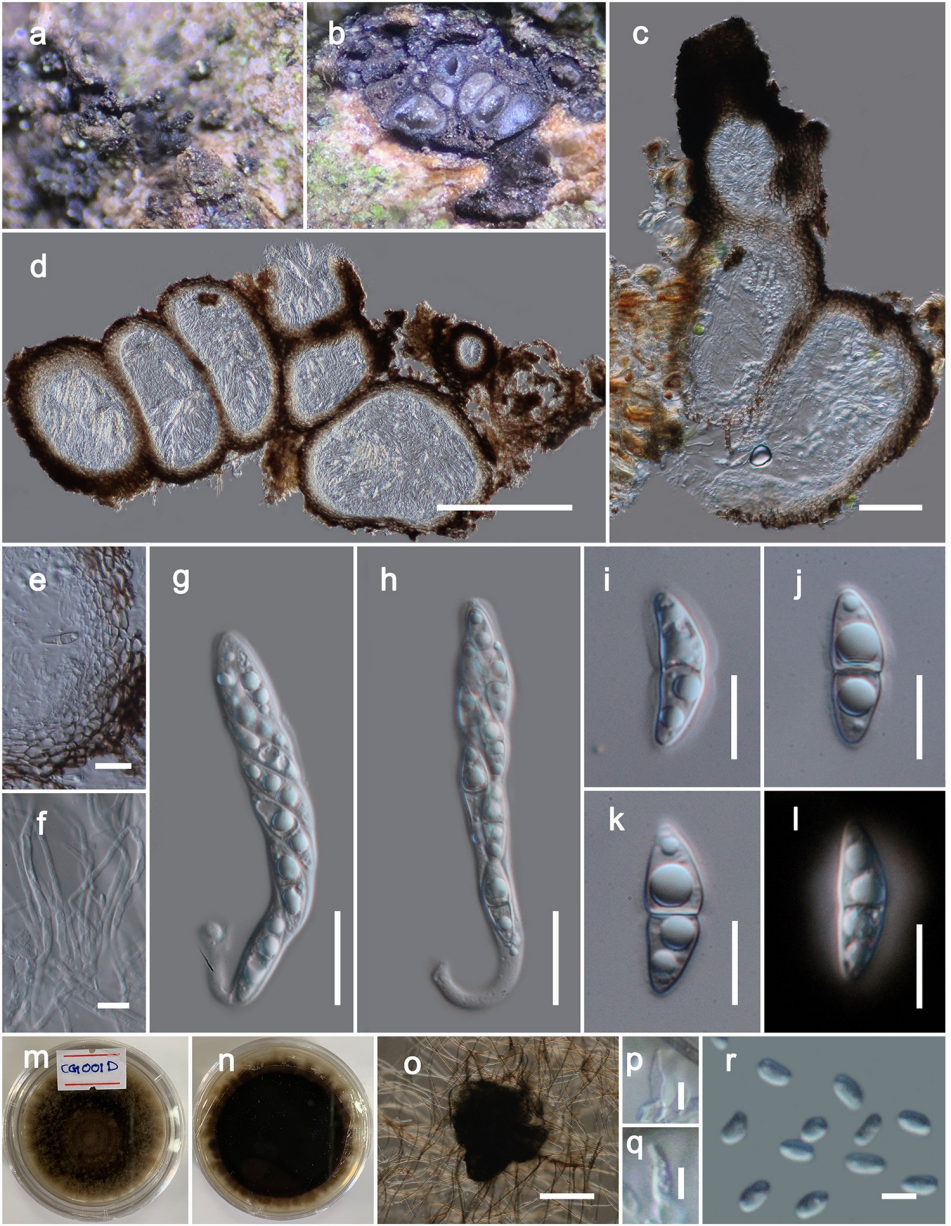
*Brunneofusispora sinensis* (MFLU 20–0093). **a** Ascomata observed on host substrate. b–d Vertical sections through ascomata. e Cells of peridium. f Pseudoparaphyses. g, h Asci. i–l Ascospores (Note the sheath stained with Indian Ink in l). m, n Colonies on PDA (n from the bottom). o Squashed conidiomata. p, q Conidiogenous cells. r Conidia. Scale bars: 50 μm (c), 200 μm (d), 20 μm (e, g, h), 10 μm (f, j–o), 100 μm (o), 5 μm (p–r).

***Brunneofusispora sinensis*** S.K. Huang & K.D. Hyde, Fungal Diversity 95: 38 (2019). Fig 5

[urn:lsid:indexfungorum.org:names:555600]

Facesoffungi number: FoF04863

*Saprobic* on dead wood or twigs. **Sexual morph**. *Ascomata* 200–300 μm high, 150–300 μm diam. ($\bar{\text{x}}=255.4\times 208.1$ μm, n = 10), perithecial, solitary to scattered, immersed, eventually erumpent, globose to subglobose, uni to multi-loculate, glabrous, dark brown to black, ostiolate. *Ostioles* 80–150 μm long, 40–70 μm diam. ($\bar{\text{x}}=117.2\times 54.6$ μm, n = 5), papillate, central. *Peridium* 20–30 μm wide, equally thick-walled, composed of blackened cells, arranged in a *textura angularis*. *Hamathecium* composed of numerous, 1.5–2.5 μm wide, filamentous, septate pseudoparaphyses. *Asci* 60–100 × 10–14 μm (x = 82.4 × 12.3 μm, n = 20), 8-spored, bitunicate, fissitunicate, cylindric-clavate to clavate, pedicellate, rounded at the apex, with an ocular chamber. *Ascospores* 16–20 × 6–8 μm (x = 18.5 × 6.4 μm, n = 30), overlapping biseriate, hyaline, broadly fusiform, 1-septate, deeply constricted at the septum, smooth-walled, with large guttules, surrounded by a thick mucilaginous sheath. **Asexual morph**. Coelomycetous. *Conidiomata* 120–160 μm high, 80–120 μm diam. ($\bar{\text{x}}=144.1\times 107.4$ μm, n = 5), solitary, superficial, globose to sub-globose, unilocular, dark brown. *Conidiomata wall* composed of thick-walled, very dark brown cells of *textura angularis*. *Conidiophores* reduced to conidiogenous cells. *Conidiogenous cells* 6–7.5 μm long, 2.5–3 μm wide ($\bar{\text{x}}=6.7\times 2.7$ μm, n = 10), enteroblastic, phialidic, ampulliform, doliiform or cylindrical, discrete, indeterminate, hyaline, smooth-walled. *Conidia* 3–4 μm × 1.9–2.5 μm ($\bar{\text{x}}=3.6\times 1.99$ μm, n = 25), mostly cylindrical or ovoid, 1-celled, hyaline, smooth, guttulate.

Culture characteristics: On PDA, colonies reached up to 40 mm diam after 15 d at 18 °C. Colony dense, circular, slightly raised at the center and raised at the margin, surface smooth, with serrate edge, floccose to fluffy, greenish grey to brown from the top and reverse dark brown, sporulated after 15–18 weeks.

Material examined: CHINA, Yunnan Province, Heilongtan, Kunming Institute of Botany, 25.137711° N, 102.745185° E, on living branches of *Magnolia denudata* (Magnoliaceae), 02 February, 2019, Dhanushka N. Wanasinghe, DWCG001D (MFLU 20–0093), living cultures MFLUCC 20–0016, KUMCC 20–0183.

Notes: In this study we have acquired DNA from the mycelium of a sexual morph and in multi-gene phylogeny, our novel strain and the *Brunneofusispora sinensis* group in a monophyletic clade (subclade B1, Fig 1). Even though B1 is not strongly supported, there were only three bp differences (not including gaps) in the comparison of the 525 nucleotides across the ITS regions. Morphological characteristics *i*.*e*. asci and ascospores, are not significantly different to each other in shape or dimensions [57]. Therefore, we identify our collection as *Brunneofusispora sinensis*. We observed its asexual morph from the culture and therefore we amended the generic and species descriptions herein. Nevertheless, it is worthy to remark that we note the ascospores of our new isolate were remaining hyaline at maturity whereas brownish in the holotype of *Brunneofusispora sinensis*.

## Discussion

The genus *Shearia* is one of the ‘most distinctive’ genera in coelomycetes [42]. Its morphology is highly conspicuous and easy to distinguish from other known dematiaceous coelomycetes [35, 42]. Several studies have discussed the morphology of this genus [35, 42]. [42, 43 and 45] reported the sexual morph of *Shearia* as *Pleomassaria*. However, *Pleomassaria maxima*, which was reported as the sexual morph of *Shearia formosa* [45] and *Shearia fusa* [43], has been transferred to *Splanchnonema maximum* (Ellis & Everh.) M.E. Barr (1993). [58] showed that *Pleomassaria s*. *str*. grouped in *Prosthemium* Kunze in their phylogenetic analyses. Based on this result, [59, 60] proposed to reduce *Pleomassaria* under *Prosthemium*. Furthermore, *Splanchnonema s*. *str*. has been accepted as a well-established genus in *Pleomassariaceae* [38, 56]. Hence, we assume that taxa linked to *Shearia* (Shear 1902) such as *Camarosporium magnoliae* [36, 42, 45], are not related to *Prosthemium* (= *Pleomassaria*) *s*. *str*. or *Splanchnonema s*. *str*., which are placed in *Pleomassariaceae*. This assumption has been confirmed by our phylogenetic analyses, placing *Shearia s*. *str*. groups in Longiostiolaceae, whereas *Splanchnonema* and *Prosthemium* strains are phylogenetically apart (Fig 1). Currently, *Pleomassaria magnoliae* (Shear 1902) and *Splanchnonema maximum* [36, 45] are lacking DNA sequencing to confirm their placements in *Pleosporales*. Consequently, doubts exist regarding the sexual morph of *Shearia*. Since both sexual morphs were collected from *Magnola* spp., we conclude that further collections from this host are necessary.

The distribution of *Magnolia* is variable but concentrated particularly around temperate and tropical South East and East Asia. They are treasured around the world as ornamental trees due to their attractive flowers and foliage and are used as timber and medicine by local and international communities. However, 48% of all *Magnolia* species are endangered and facing habitat loss [61]. The Kunming Botanical Garden functions as an ex-situ conservation for endangered, endemic and economically important plant species native to the Yunnan Plateau and the southern Hengduan Mountains [6]. Micro-fungi on *Magnolia* have been studied for decades around the world, and so far over 1000 fungal species have been reported on this host [62]. However, only 46 fungal species have been reported from China, across just 14 *Magnolia* species (*M*. *alba*, *M*. *albosericea*, *M*. *candolii*, *M*. *coco*, *M*. *cylindrica*, *M*. *delavayi*, *M*. *denudata*, *M*. *grandiflora*, *M*. *liliiflora*, *M*. *officinalis*, *M*. *paenetalauma*, *M*. *soulangeana*, *M*. *stellata* and *M*. *zenii*) [62]. This study highlights the Kunming Botanical Garden as an ideal locale to conduct diverse research on the micro-fungal occurrences on *Magnolia* as it is a repository of a great number of *Magnolia* species.

## Supporting information

S1 Table Taxa used in the phylogenetic analyses and their corresponding GenBank numbers. The newly generated sequences are indicated in bold.
(DOC)
