# Supplementary material for: Taxonomic novelties in Magnolia-associated pleosporalean fungi in the Kunming Botanical Gardens (Yunnan, China)
Source: PLoS One. 2020 Jul 13;15(7):e0235855. doi: 10.1371/journal.pone.0235855 (PMC7357747; doi:10.1371/journal.pone.0235855)
Supplement: S1 Table — The newly generated sequences are indicated in bold. (DOC) [file pone.0235855.s001.doc]

| **Species** | **Strain no** | **GenBank Accession no** | | | |
| --- | --- | --- | --- | --- | --- |
|  |  | **LSU** | **SSU** | **TEF** | **RPB2** |
| *Aigialus grandis* | BCC 20000 | GU479775 | GU479739 | GU479839 | GU479814 |
| *Aigialus mangrovis* | BCC 33563 | GU479776 | GU479741 | GU479840 | GU479815 |
| *Aigialus parvus* | BCC 18403 | GU479777 | GU479742 | GU479841 | GU479816 |
| *Aigialus rhizophorae* | BCC 33572 | GU479780 | GU479745 | GU479844 | GU479819 |
| *Amniculicola lignicola* | CBS 123094 | EF493861 | EF493863 | GU456278 | EF493862 |
| *Angustimassarina acerina* | MFLUCC 14 0505 | KP888637 | KP899123 | KR075168 |  |
| *Angustimassarina populi* | MFLUCC 13-0034 | KP888642 | KP899128 | KR075164 |  |
| *Angustimassarina quercicola* | MFLUCC 14-0506 | KP888638 | KP899124 | KR075169 |  |
| *Anteaglonium abbreviatum* | GKM925.1 | GQ221877 |  | GQ221924 |  |
| *Anteaglonium parvulum* | C 024 | KU922915 | KU922916 | KU922921 |  |
| *Aquasubmersa japonica* | KT2813 | LC061586 | LC061581 | LC194383 | LC194420 |
| *Aquasubmersa japonica* | KT2862 | LC061587 | LC061582 | LC194384 | LC194421 |
| *Aquasubmersa japonica* | KT2863 | LC061588 | LC061583 | LC194385 | LC194422 |
| *Ascochyta coronillae-emeri* | MFLUCC 13-0820 | MH069667 | MH069673 |  | MH069679 |
| *Astrosphaeriella fusispora* | MFLUCC 10-0555 | KP057604 | KT955443 | KT955425 | KT955413 |
| *Astrosphaeriella neofusispora* | MFLUCC 11-0161 | KT955463 | KT955444 | KT955426 | KT955418 |
| *Atrocalyx acutisporus* | HHUF 30504H | LC194341 | LC194299 | LC194386 | LC194423 |
| *Atrocalyx oxysporus* | HHUF 29084H | LC194344 | LC194302 | LC194389 | LC194426 |
| *Bambusicola bambusae* | MFLUCC 11-0614 | JX442035 | JX442039 | KP761722 | KP761718 |
| *Bambusistroma didymosporum* | MFLUCC 13-0862 | KP761731 | KP761738 | KP761728 | KP761721 |
| *Biatriospora mackinnonii* | CBS 110022 | GQ387613 | GQ387552 |  | KF015703 |
| *Biatriospora mackinnonii* | CBS 674.75 | GQ387614 | GQ387553 | KF407985 | KF015704 |
| *Biatriospora marina* | CY 1228 | GQ925848 | GQ925835 | GU479848 | GU479823 |
| *Biatriospora peruviensis* | CCF 4485 | LN626683 | LN626677 | LN626671 | LN626665 |
| *Biatriospora sp.* | E8502C | LN626680 |  | LN626672 |  |
| *Botryosphaeria dothidea* | CBS 115476 | NG_027577 | DQ677998 | DQ767637 | DQ677944 |
| *Brevicollum hyalosporum* | MAFF 243400 | NG_058715 | NG_065123 | LC271245 | LC271249 |
| *Brevicollum versicolor* | HHUF 30591 | NG_058716 | LC271237 | LC271246 | LC271250 |
| *Brunneofusispora sinensis* | KUMCC 17-0030 | MH393557 | MH393556 | MH395329 |  |
| ***Brunneofusispora sinensis*** | **MFLUCC 20-0016** | **MT159624** | **MT159636** | **MT159607** | **MT159613** |
| *Camarosporidiella caraganicola* | MFLUCC 14-0887 | MF434209 | MF434297 | MF434385 |  |
| *Crassiparies quadrisporus* | HHUF 30409 | NG_059028 | NG_061267 | LC271248 | LC271252 |
| *Crassiperidium octosporum* | KT 2144 | LC373108 | LC373084 | LC373120 | LC373132 |
| *Crassiperidium octosporum* | KT 2894 | LC373109 | LC373085 | LC373121 | LC373133 |
| *Crassiperidium octosporum* | KT 3008 | LC373110 | LC373086 | LC373122 | LC373134 |
| *Crassiperidium quadrisporum* | KT 2798-1 | LC373118 | LC373094 | LC373130 | LC373142 |
| *Crassiperidium quadrisporum* | KT 2798-2 | LC373119 | LC373095 | LC373131 | LC373143 |
| *Cryptoclypeus ryukyuensis* | HHUF 30510P | LC194346 | LC194304 | LC194391 | LC194428 |
| *Cryptocoryneum akitaense* | KT3019 | LC194348 | LC194306 | LC096136 | LC194430 |
| *Cryptocoryneum brevicondensatum* | yone152 | LC194349 | LC194307 | LC096137 | LC194431 |
| *Cryptocoryneum longicondensatum* | KT2913 | LC194360 | LC194318 | LC096148 | LC194442 |
| *Cyclothyriella rubronotata* | CBS 121892 | KX650541 |  | KX650516 | KX650571 |
| *Cyclothyriella rubronotata* | CBS 141486 | KX650544 | NG_061252 | KX650519 | KX650574 |
| *Cyclothyriella rubronotata* | TR1 | KX650542 |  | KX650517 | KX650572 |
| *Cyclothyriella rubronotata* | TR3 | KX650543 |  |  | KX650573 |
| *Cyclothyriella rubronotata* | TR9a | KX650545 |  |  |  |
| *Decaisnella formosa* | BCC 25616 | GQ925846 | GQ925833 | GU479851 | GU479825 |
| *Decaisnella formosa* | BCC 25617 | GQ925847 | GQ925834 | GU479850 | GU479824 |
| *Delitschia chaetomioides* | SMH 3253.2 | GU390656 |  | GU327753 |  |
| *Delitschia winteri* | AFTOLID 1599 | DQ678077 | DQ678026 | DQ677922 | DQ677975 |
| *Diatrype disciformis* | AFTOL-ID 927 | DQ470964 | DQ471012 | DQ471085 | DQ470915 |
| *Dothidotthia negundinicola* | MFLUCC 16-1157 | MK751815 | MK751760 | MK908015 | MK920235 |
| *Fusculina eucalypti* | CBS 120083 | DQ923531 |  |  |  |
| *Fusculina eucalyptorum* | CBS 145083 | MK047499 |  |  |  |
| *Gordonomyces mucovaginatus* | CBS 127273 | NG_057941 | |  |  |
| *Graphostroma platystoma* | CBS 270.87 | DQ836906 | DQ836900 | DQ836915 | DQ836893 |
| *Guttulispora crataegi* | MFLUCC 14-0993 | KP888640 | KP899126 | KR075162 |  |
| *Halotthia posidoniae* | BBH 22481 | GU479786 | GU479752 |  |  |
| *Hermatomyces tectonae* | MFLUCC 14 1140 | KU764695 | KU712465 | KU872757 | KU712486 |
| *Hermatomyces thailandica* | MFLUCC 14 1143 | KU764692 | KU712468 | KU872754 | KU712488 |
| *Herpotrichia diffusa* | AFTOL-ID 1588 | DQ678071 | DQ678019 | DQ677915 | DQ677968 |
| *Herpotrichia juniperi* | AFTOL-ID 1608 | DQ678080 | DQ678029 | DQ677925 | DQ677978 |
| *Hobus wogradensis* | CBS 141484 | KX650546 | KX650508 | KX650521 | KX650575 |
| *Hypsostroma caimitalense* | GKM 1165 | GU385180 |  |  |  |
| *Hypsostroma saxicola* | SMH 5005 | GU385181 |  |  |  |
| *Katumotoa bambusicola* | KT 1517a | AB524595 | AB524454 | AB539108 | AB539095 |
| *Lentimurispora urniformis* | MFLUCC 18-0497 | MH179144 | MH179160 | MH188055 |  |
| *Leptosphaerulina australis* | CBS 317.83 | GU301830 | GU296160 | GU349070 | GU371790 |
| *Ligninsphaeria jonesii* | MFLUCC 15-0641 | NG_059642 | |  |  |
| *Ligninsphaeria jonesii* | GZCC 15-0080 | KU221038 |  |  |  |
| *Lindgomyces cinctosporae* | R56 | AB522431 | AB522430 |  |  |
| *Lindgomyces ingoldianus* | ATCC 200398 | AB521736 | AB521719 |  |  |
| *Lindgomyces rotundatus* | KT 1096 | AB521740 | AB521723 |  |  |
| *Longiostiolum tectonae* | MFLUCC 12-0562 | KU764700 | NG_061231 | |  |
| *Lophiostoma arundinis* | CBS 621.86 | DQ782384 | DQ782383 | DQ782387 | DQ782386 |
| *Lophiostoma crenatum* | AFTOLID 1581 | DQ678069 | DQ678017 | DQ677912 | DQ677965 |
| *Lophium mytilinum* | CBS 269.34 | DQ678081 | DQ678030 | DQ677926 |  |
| *Massaria gigantispora* | WU 30521 | HQ599397 | HQ599447 | HQ599337 |  |
| *Massaria inquinans* | WU 30527 | HQ599402 | HQ599444 | HQ599342 | HQ599460 |
| *Massarina eburnea* | CBS 473.64 | GU301840 | GU296170 | GU349040 | GU371732 |
| *Massarina rubi* | CBS 691.95 | FJ795453 | GU456301 |  | FJ795470 |
| *Massarina rubi* | MUT 4323 | KF636772 |  |  |  |
| *Massarina rubi* | MUT 4887 | KP671721 | KT587318 |  |  |
| *Mauritiana rhizophorae* | BCC 28866 | GU371824 | GU371832 | GU371817 |  |
| *Melanomma pulvispyrius* | CBS 124080 | GU456323 | GU456302 | GU456265 | GU456350 |
| *Multilocularia bambusae* | MFLUCC 11-0180 | NG_059654 | NG_061229 | KU705656 |  |
| *Murilentithecium clematidis* | MFLUCC 14-0561 | KM408758 | KM408760 | KM454444 | KM454446 |
| *Murispora hawksworthii* | MFLUCC 14-0918 | KT709180 | KT709187 | KT709192 |  |
| *Mytilinidion rhenanum* | CBS 135.34 | FJ161175 | FJ161136 | FJ161092 |  |
| *Neoaquastroma guttulatum* | MFLUCC 14-0917 | KX949740 | KX949741 | KX949742 |  |
| *Neocucurbitaria rhamni* | CBS 142391 | MF795775 | MF795838 | MF795863 | MF795817 |
| *Neooccultibambusa chiangraiensis* | MFLUCC 12-0559 | KU764699 | KU712458 | KU872761 |  |
| *Neooccultibambusa jonesii* | MFLUCC 16 643 | NG_059741 | NG_062422 | |  |
| *Neooccultibambusa pandanicola* | KMUCC 17-0179 | MG298940 | MG298942 | MG298943 | MG298944 |
| *Neooccultibambusa thailandensis* | MFLUCC 16-0274 | MH260308 | MH260348 | MH412780 | MH412758 |
| *Nigrograna antibiotica* | CCF 4378T | KF925327 | KF925328 | JX570934 | LN626661 |
| *Nigrograna cangshanensis* | MFLUCC 15-0253 | NG_059778 | NG_063630 | KY511066 |  |
| *Nigrograna carollii* | CCF 4884 | LN626682 | LN626674 | LN626668 | LN626662 |
| *Nigrograna fuscidula* | MF1 | KX650547 | KX650509 | KX650522 | KX650576 |
| *Nigrograna locuta-pollinis* | LC11685 | MF939583 |  | MF939613 | MF939610 |
| *Nigrograna mackinnonii* | E5202H | LN626681 | LN626678 | LN626673 | LN626666 |
| ***Nigrograna magnoliae*** | **MFLUCC 20-0020** | **MT159622** | **MT159634** | **MT159605** | **MT159611** |
| ***Nigrograna magnoliae*** | **MFLUCC 20-0021** | **MT159623** | **MT159635** | **MT159606** | **MT159612** |
| *Nigrograna mycophila* | MF1 | KX650547 | KX650509 | KX650522 | KX650576 |
| *Nigrograna mycophila* | TDK | KX650555 | KX650510 | KX650528 | KX650577 |
| *Nigrograna norvegica* | CBS 141485 | KX650556 | NG_063066 | | KX650578 |
| *Nigrograna obliqua* | CBS 141475 | KX650558 | KX650512 | KX650530 | KX650579 |
| *Nigrograna obliqua* | MF2 | KX650560 |  | KX650531 | KX650580 |
| *Nigrograna rhizophorae* | MFLUCC 18-0397 | MN420686 |  | MN077063 | MN431490 |
| *Nigrograna rhizophorae* | MCD185 | MN017845 |  | MN077064 | MN431489 |
| *Nigrograna thailandica* | MFLUCC 17-2663 | MK762716 | MK762704 |  |  |
| *Nigrograna thymi* | MFLU 17-0497 | NG_064431 | NG_065679 | KY775578 |  |
| *Nigrograna yasuniana* | YU.101026T | LN626684 | LN626676 | LN626670 | LN626664 |
| *Occultibambusa aquatica* | MFLUCC 11-0006 | KX698110 | KX698112 |  |  |
| *Occultibambusa bambusae* | MFLUCC 11-0394 | KU863113 |  |  | KU940171 |
| *Occultibambusa bambusae* | MFLUCC 13-0855 | KU863112 | KU872116 | KU940193 | KU940170 |
| *Occultibambusa chiangraiensis* | MFLUCC 16-0380 | KX655546 | NG_062421 | KX655561 | KX655566 |
| *Occultibambusa fusispora* | MFLUCC 11-0127 | KU863114 |  | KU940195 | KU940172 |
| *Occultibambusa jonesii* | GZCC 16-0117 | NG_066381 | NG_065104 | KY814756 | KY814758 |
| *Occultibambusa maolanensis* | GZCC 16-0116 | KY628323 | KY628325 | KY814757 | KY814759 |
| *Occultibambusa pustula* | MFLUCC 11-0502 | KU863115 | KU872118 |  |  |
| *Ohleria modesta* | CBS 141480 | KX650563 | KX650513 | KX650534 | KX650583 |
| *Ohleria modesta* | WU 36870 | KX650562 |  | KX650533 | KX650582 |
| *Paradictyoarthrinium diffractum* | MFLUCC13-0466 | KP744498 | KP753960 |  |  |
| *Paradictyoarthrinium hydei* | MFLUCC 17-2512 | MG747497 | NG_065757 | | MG780232 |
| *Paradictyoarthrinium tectonicola* | MFLUCC 13-0465 | KP744500 | KP753961 |  |  |
| *Parathyridaria ramulicola* | CBS 141479 | KX650565 | KX650514 | KX650536 | KX650584 |
| *Phaeoseptum terricola* | MFLUCC 10-0102 | MH105779 | MH105780 | MH105781 | MH105782 |
| *Phyllosticta ampelicida* | CBS 111645 | DQ377876 | EU673223 |  |  |
| *Pleomassaria siparia* | CBS 279.74 | DQ678078 | DQ678027 | DQ677923 | DQ677976 |
| *Prosthemium canba* | JCM 16966 | AB553760 | AB553646 |  |  |
| *Prosthemium orientale* | JCM 12841 | AB553748 | AB553641 |  |  |
| *Pseudoastrosphaeriella bambusae* | MFLUCC 11-0205 | KT955475 | KT955455 | KT955437 | KT955414 |
| *Pseudoastrosphaeriellaceae longicolla* | MFLUCC 11-0171 | KT955476 |  | KT955438 | KT955420 |
| *Pseudoastrosphaeriellaceae thailandensis* | MFLUCC 10-0553 | KT955477 | KT955456 | KT955439 | KT955411 |
| *Pseudoastrosphaeriellopsis kaveriana* | PUFD33 | MG947595 | MG947598 | MG968955 | MG948547 |
| *Pseudoberkleasmium acaciae* | MFLUCC 17-2590 | NG_066316 | NG_065782 | MK360073 |  |
| *Pseudoberkleasmium chiangmaiense* | MFLUCC 17-1809 | MK131260 |  | MK131261 |  |
| *Pseudoberkleasmium pandanicola* | KUMCC 17-0178 | MH260304 | MH260344 |  |  |
| *Pseudocoleodictyospora sukhothaiensis* | MFLUCC 12-0554 | KU764710 | KU712471 |  | KU712493 |
| *Pseudocoleodictyospora tectonae* | MFLUCC 12-0385 | KU764709 | KU712461 |  | KU712491 |
| *Pseudocoleodictyospora tectonae* | MFLUCC 12-0387 | KU764704 | KU712462 |  | KU712492 |
| *Pseudolophiotrema elymicola* | KT1450 | LC194381 | LC194339 | LC194418 | LC194473 |
| *Pseudomassariosphaeria bromicola* | MFLUCC 15-0031 | KT305994 | KT305996 | KT305999 |  |
| *Roussoella hysterioides* | CBS 546.94 | KF443381 | AY642528 | KF443399 | KF443392 |
| *Roussoella pustulans* | MAFF 239637 | AB524623 | AB524482 | AB539116 | AB539103 |
| *Salsuginea ramicola* | KT 2597.1 | GU479800 | GU479767 | GU479861 | GU479833 |
| *Salsuginea ramicola* | KT 2597.2 | GU479801 | GU479768 | GU479862 | GU479834 |
| *Seriascoma didymospora* | MFLUCC 11-0179 | KU863116 | KU872119 | KU940196 | KU940173 |
| *Seriascoma didymospora* | MFLUCC 11-0194 | KU863117 | KU872120 | KU940197 | KU940174 |
| ***Shearia formosa*** | **MFLUCC 20-0017** | **MT159619** | **MT159631** | **MT159602** | **MT159608** |
| ***Shearia formosa*** | **MFLUCC 20-0018** | **MT159621** | **MT159633** | **MT159604** | **MT159610** |
| ***Shearia formosa*** | **MFLUCC 20-0019** | **MT159620** | **MT159632** | **MT159603** | **MT159609** |
| *Sigarispora arundinis* | JCM 13550 | AB618998 | AB618679 | LC001737 |  |
| *Sordaria fimicola* | AFTOL-ID 216 | FR774289 | AH007748 | DQ518175 | DQ368647 |
| *Splanchnonema platani* | CBS 222.37 | KR909316 | KR909318 | KR909319 | KR909322 |
| *Sporormiella minima* | Lundqvist17212 a | DQ678056 | DQ678003 | DQ677897 | DQ677950 |
| *Stemphylium vesicarium* | CBS 191.86 | MH873624 | GU238232 |  | KC584471 |
| *Striatiguttula nypae* | MFLUCC 17-2517 | MK035993 | MK035978 | MK034433 | MK034441 |
| *Striatiguttula nypae* | MFLUCC 18-0265 | MK035992 | MK035977 | MK034432 | MK034440 |
| *Striatiguttula phoenicis* | MFLUCC 18-0266 | MK035995 | MK035980 | MK034435 | MK034442 |
| *Sulcosporium thailandica* | MFLUCC 12-0004 | KT426563 | KT426564 |  |  |
| *Teichospora rubriostiolata* | TR5 | KU601589 |  | KU601606 | KU601598 |
| *Teichospora rubriostiolata* | TR7 | KU601590 |  | KU601609 | KU601599 |
| *Teichospora trabicola* | C134 | KU601591 |  | KU601601 | KU601600 |
| *Tetraplosphaeria sasicola* | JCM 13167 | AB524631 | AB524490 |  |  |
| *Tetraplosphaeria yakushimensis* | KT 1906 | AB524632 | AB524491 |  |  |
| *Thyridaria broussonetiae* | TB1 | KX650569 | KX650515 | KX650539 | KX650586 |
| *Thyrostroma lycii* | MFLUCC 16-1170 | MK751824 | MK751769 | MK908024 | MK920241 |
| *Torula herbarum* | CBS 111855 | KF443386 | KF443391 | KF443403 | KF443396 |
| *Torula hollandica* | CBS 220.69 | KF443384 | KF443389 | KF443401 | KF443393 |
| *Tzeanania taiwanensis* | NTUCC 17-005 | MH461120 | MH461126 | MH461130 | MH461128 |
| *Ulospora bilgramii* | CBS 101364 | KF443382 |  | KF443399 | KF443392 |
| *Verruculina enalia* | BCC 18401 | GU479802 | GU479770 | GU479863 | GU479835 |
| *Verruculina enalia* | BCC 18402 | GU479803 | GU479771 | GU479864 | GU479836 |
| *Versicolorisporium triseptatum* | HHUF 28815 | AB330081 | AB524501 |  |  |
| *Westerdykella cylindrica* | AFTOL-ID 1037 | AY004343 | AY016355 | DQ497610 |  |
| *Westerdykella ornata* | CBS 379.55 | GU301880 | GU296208 | GU349021 | GU371803 |
| *Wicklowia aquatica* | AF289-1 | GU045446 | GU266232 |  |  |
| *Wicklowia aquatica* | F76-2 | GU045445 |  |  |  |
| *Zopfia rhizophila* | CBS 207.26 | DQ384104 |  |  |  |
